# *In Vitro* Synergistic Inhibitory Activity of Natural Alkaloid Berberine Combined with Azithromycin against Alginate Production by *Pseudomonas aeruginosa* PAO1

**DOI:** 10.1155/2022/3858500

**Published:** 2022-09-10

**Authors:** Zhijing Zhao, Mengyu Guo, Xiaona Xu, Yue Hu, Dongmei Liu, Chunxia Wang, Xinwei Liu, Yongwei Li

**Affiliations:** ^1^Henan University of Chinese Medicine, Zhengzhou, China; ^2^Henan Province Hospital of Traditional Chinese Medicine, Zhengzhou, China; ^3^The Second Affiliated Hospital of Henan University of Chinese Medicine, Zhengzhou, China; ^4^The Key Laboratory of Pathogenic Microbes &Antimicrobial Resistance Surveillance of Zhengzhou, Zhengzhou, China; ^5^Henan Engineering Research Center for Identification of Pathogenic Microbes, Zhengzhou, China; ^6^Henan Provincial Key Laboratory of Antibiotics-Resistant Bacterial Infection Prevention & Therapy with Traditional Chinese Medicine, Zhengzhou, China

## Abstract

**Background:**

Berberine (BER) is a natural isoquinoline alkaloid which extensively been applied to treat bacterial infection in TCM for a long time. Alginate is an important component of *Pseudomonas aeruginosa* biofilm. Herein, we investigated the effects of berberine and azithromycin (AZM) on alginate in the biofilm of *P. aeruginosa* PAO1.

**Methods:**

The MIC and synergistic activity of BER and AZM against PAO1 were determined using the micro broth dilution and checkerboard titration methods, respectively. The effect of BER on PAO1 growth was evaluated using a time-kill assay. Moreover, the effects of BER, AZM, and a combination of both on PAO1 biofilm formation, kinesis, and virulence factor expression were evaluated at subinhibitory concentrations. The alginate content in the biofilm was detected using ELISA, and the relative expression of alginate formation-related genes *algD*, *algR*, and *algG* was detected by qRT-PCR.

**Results:**

Simultaneous administration of berberine significantly reduced the MIC of azithromycin, and berberine at a certain concentration inhibited PAO1 growth. Moreover, combined berberine and azithromycin had synergistic effects against PAO1, significantly reducing biofilm formation, swarming, and twitching motility, and the production of virulence factors. The relative expression of alginate-related regulatory genes *algG*, *algD*, and *algR* of the combined treatment group was significantly lower than that of the control group.

**Conclusion:**

In summary, berberine and azithromycin in combination had a significant synergistic effect on the inhibition of alginate production by *P. aeruginosa*. Further molecular studies are in great need to reveal the mechanisms underlying the synergistic activity between berberine and azithromycin.

## 1. Introduction

*Pseudomonas aeruginosa* (*P. aeruginosa*) is an opportunistic, environmental pathogen that has become one of the most prevalent causes of healthcare-associated infection in recent years, due to its unique biofilm lifestyle [1]. Biofilm produced by *P. aeruginosa* enables it, especially in patients with cystic fibrosis (CF), to increase its tolerance to antibiotics and promote the persistence of infection [2]. A biofilm is composed of 15% attached bacterial cells and 85% extracellular matrix formed by bacterially produced structural elements, comprising extracellular polysaccharides (EPS) as well as proteins, lipids, and polymers, such as e-DNA [3, 4]. Alginate secreted by *P. aeruginosa* is a sort of important polymeric substance that facilitates the non-specific attachment of bacteria to surfaces to expedite biofilm formation and render the bacteria resistant to antibiotics and host immune responses [5].

The overproduction of alginate was first associated with mucoid *P. aeruginosa* strains isolated from the lung of chronically infected CF patients but is rarely isolated from other types of infections [6, 7]. In patients with chronic lower respiratory tract infection, researchers employed Whole Genome Shotgun Sequencing to screen gene mutations that contribute to the long-term survival of *P. aeruginosa* in the lung of patients with CF. Over time, genome analysis revealed that strains which underwent loss-of-function mutations in the *mucA* gene resulted in the mucoid conversion of *P. aeruginosa* and subsequent excessive production of alginate, which contributes to lung obstruction in CF patients [8]. Moreover, with the assistance of alginate, mucoid *P. aeruginosa* can inhibit macrophage phagocytosis [9] and alveolar macrophage apoptotic cell clearance [10], which involves protecting mucoid *P. aeruginosa* against the host immune system.

Adjuvant therapy of Macrolide (ML) is of great importance in the maintenance therapy of patients with chronic CF infection [11]. Previous studies have shown that long-term, low-dose ML antibiotic therapy is beneficial to CF patients, and azithromycin (AZM) administration in this manner is part of the standard therapy regimen in most CF centers, globally [12, 13]. However, the bactericidal activity of AZM against *P. aeruginosa* is relatively low and needs to combine with other drugs to reduce its concentration to effective concentration [14].

Studies have shown that various natural preparations, such as fungal metabolites [15, 16] and plant extracts [17, 18], possess anti-biofilm and -virulence effects against *P. aeruginosa*. Berberine (BER) is a natural isoquinoline alkaloid mainly derived from *Rhizoma Coptidis, Cortex Phellodendri*, and *Radix Scutellariae*. According to the 2020 edition of Pharmacopoeia of the People's Republic of China [19] and Chinese Herb Dictionary [20], the main pharmacologic effectiveness of *Rhizoma Coptidis, Cortex Phellodendri*, and *Radix Scutellariae* are heat-clearing and damp-drying, fire-purging for toxin-removing, and the traditional use of these herbs is to treat bacterial infection such as bacterial dysentery and acute lower respiratory infection caused by pathogenic microorganisms. As the main compound of the herbs, BER is generally considered inexpensive, safe, and well-known for its broad antibacterial activities and used in the treatment of microbial infections for a long history in Traditional Chinese Medicine (TCM). In recent years, a mass of animal and clinical data [21–23] have shown that BER presents a broad spectrum of pharmacological effects, including anti-inflammatory, antibacterial, and antitumor, as well as blood sugar- and lipid-lowering, among others, and is used to treat a series of diseases [24, 25]. As a broad-spectrum antibacterial, heat-clearing, and detoxifying drug, BER shows an inhibitory effect on kinds of gram-positive and -negative bacteria, *in vitro* [26]. However, present research concentrates on the effect of BER on *P. aeruginosa* biofilm, and alginate of mucoid *P. aeruginosa* biofilm, specifically, is limited. In this study, we aimed to explore the effects of various combinations of BER and AZM on alginate—a key component of PAO1 biofilm—as well as biofilm, virulence factors, and motility of *P. aeruginosa*, to identify a new approach to the clinical treatment of *P. aeruginosa* biofilm infection.

## 2. Materials and Methods

### 2.1. Bacterial Strains

The *P. aeruginosa* strain PAO1 (ATCC15692/DSM22644, Catalog Number: Bio-82076) was purchased from Beijing Baiou Bowei Biotechnology Co., Ltd. Bacteria were maintained at -80°C in Luria-Bertani (LB) broth containing 20% (v/v) glycerol. The bacterial culture was revived on LB agar plates, and a single colony was inoculated in LB broth at 37°C, followed by incubation in a shaker at 220 rpm, for 20 h.

### 2.2. Materials

Berberine hydrochloride (Shanghai Sangon Bioengineering Co., Ltd., Shanghai, China, E205BA0008) was prepared according to the standards set by the Clinical and Laboratory Standards Institute (CLSI) to a final concentration of 20 mg/mL. AZM was used at concentrations of 0.25–1024 *μ*g/mL. Anti-alginate monoclonal antibodies (StressMarq Biosciences Inc., Victoria, BC, Canada, SMC-208) was used as primary antibody, and anti-mouse secondary antibody (Shanghai Sangon Bioengineering Co., Ltd., Shanghai, China, D110087-0025) was used.

### 2.3. Determination of MIC of BER and AZM

The Minimum Inhibitory Concentration (MIC) for both BER and AZM was evaluated through broth microdilution (BMD), according to the CLSI standards document M07-A10. Multiple dilutions of BER and AZM were prepared in a 96-well plate to a volume of 100 *μ*L per well. The final concentrations of BER and AZM were 39.1 and 1.0 *μ*g/mL, respectively. Thereafter, 100 *μ*L of bacterial suspension was added to each well, to a bacterial concentration of approximately 5 × 10^5^ CFU/mL. In control cultures, bacteria were allowed to grow in Mueller Hinton (MH) broth (Haibo Bioengineering Co., Ltd., Shanghai, China, HB6231) at pH 7.2–7.4, without the addition of BER or AZM. After plates were incubated at 37°C for 24 h, MIC was defined as the lowest concentration of the tested preparation that completely inhibited visible bacterial growth in MH broth. The combined effects of BER and AZM were evaluated using checkerboard titration. The Fractional Inhibitory Concentration Index (FICI) was calculated as FICI = (combinatorial MIC BER_AZM/individual MIC BER) + (combinatorial MIC BER_AZM/individual MIC AZM), with the following results interpretation: FICI ≤ 0.5 (synergistic effects), 0.5 < FICI ≤ 1 (cumulative effects), 1 < FICI ≤ 4 (irrelevant effects), and FICI > 4 (antagonistic effects).

### 2.4. Time-Kill Assays

The effect of BER on the growth of PAO1 was evaluated using a growth curve. Briefly, a suspension of PAO1 bacteria in the logarithmic growth phase was added to the LB medium and cultured to an A600 value of 0.3. BER was added to the culture to final concentrations of 39.1, 78.2, 156.3, 312.5, and 625.0 *μ*g/mL, respectively. Control cultures were set up in the same manner, but without the addition of BER. Cultures were grown at 37°C, 200 rpm for 24 h. The A600 was measured hourly, and the experimental procedure was repeated three times for each preparation. Results were observed for 24 h, whereafter growth curves were constructed.

### 2.5. Biofilm Quantification

The biofilm production of PAO1 under static conditions was determined using crystal violet (CV) staining. To detect inhibition by subinhibitory concentrations of BER, AZM, and a combination of both on biofilm formation by *P. aeruginos*a, wells of a 96-well plate were inoculated with 100 *μ*L bacterial suspension containing sub-MICs of BER, AZM, or a combination of both. Three duplicated wells were prepared simultaneously per drug concentration, along with control wells lacking drugs. The plate was statically incubated at 37°C for 48 h to promote cell attachment and biofilm formation. Non-adherent cells in the supernatant were then removed with a pipette and the plate was gently rinsed three times with phosphate-buffered saline (PBS). Thereafter, 100 *μ*L of 1% CV was added and allowed to stain for 15 min. The plate was washed with PBS once more, the excess dye removed, and the plate air-dried. Dye absorbed by the biofilm cells was extracted by adding 100 *μ*L of glacial acetic acid at a concentration of 33% (v/v) to each well. Absorbance was measured at 570 nm using an enzyme-labelling instrument (Dana Tianjin Biological Technology Co., Ltd, Tianjin, China, 16039400). The experimental procedure was repeated three times to obtain mean absorbance values.

### 2.6. Quantification of Extracellular Polysaccharides (EPS) and Total Protein of Biofilm

EPS secreted by *P. aeruginosa* was measured using a previously described method [27]. Briefly, a 50-*μ*L sample of *P. aeruginosa* at a concentration of 1 × 10^6^ CFU/mL was inoculated in a sterile LB medium and cultured in a 90-mm glass Petri dish. Multiple concentrations of the drugs were added under sterile conditions. The same experimental protocol and bacterial concentration were applied to the control cultures, except without drug exposure. All dishes were incubated at 37°C for 48 h, with planktonic cells discarded thereafter. The biofilm that had formed at the bottom of each Petri dish was recovered by scraping with a cell scraper, after adding sterile double-distilled water to the dish. The resultant bacterial biofilm suspension was centrifuged at 4000 g at 4°C for 20 min, and the supernatant was collected. The cell precipitates were further treated with 10 mM ethylenediaminetetraacetic acid (EDTA), swirled for 10 min, and centrifuged again at 3500 g for 20 min, to extract cell-bound EPS. The collected supernatant was then mixed with an earlier batch that had been prepared similarly and stored. The combined supernatant was mixed with frozen anhydrous ethanol and incubated at ˗20°C for 1 h. Thereafter, the supernatant was centrifuged at 3500 g at 4°C for 20 min. The precipitate was collected and dissolved in sterile double-distilled water and the EPS content in the particles was determined using the phenol-sulfuric acid method.

The protocols of PAO1 biofilm total protein quantification were previously described with some modifications [27]. Briefly, PAO1 biofilms were incubated with or without BER and AZM alone and in different combination patterns at 37°C for 48 h. After incubations, the biofilms were washed gently with pre-cold sterile PBS to remove planktonic cells and adhered loosely cells. The biofilm cells were then scratched into a test tube, boiled in 5 mL 15 nM NaOH for 30 min, and centrifuged at 10,000 g for 5 min. The concentrations of total protein of each group were measured following the instructions of the BCA Protein Assay Kit (Beyotime, Shanghai, China).

### 2.7. Quantitative Determination of Pyocyanin Production and LasB Activity

The yield of pyocyanin in *P. aeruginosa* was determined as described [28]. Briefly, bacteria that had been cultured overnight were inoculated into an aseptic LB medium containing BER, AZM, or a combination of both, at multiple sub-MIC concentrations. In control cultures, no drugs were added to the medium. Both treated and control bacterial cultures were incubated at 37°C for 48 h. After incubation, microbial cultures were collected from each growth medium and centrifuged at 4000 g for 10 min. A 3-mL aliquot of chloroform and 1 mL hydrochloric acid were added to the collected supernatant, changing its color from green to yellow. The color intensity of the solution was quantified by measuring the absorbance of the solution at 520 nm, using an enzyme-labelling instrument (Dana Tianjin Biological Technology Co., Ltd, Tianjin, China, 16039400).

The elastase activity of LasB in culture supernatants was determined using the elastin-Congo red (ECR) method. Briefly, 500 *μ*L of filtered, sterilized supernatant was added to 500 *μ*L ECR buffer containing 10 mg ECR (100 mM Tris-HCl, pH 7.5). The mixture was then rotated at 37°C for 6 h. After the insoluble ECR was removed through centrifugation, absorbance was measured at 495 nm, using an enzyme-labelling instrument.

### 2.8. Swarming and Twitching Motility Assay

To study the effects of BER and AZM on motility of PAO1, the recommended approach [29] of the motility assay was adopted. Briefly, the culture medium was prepared in a 90 × 15 mm Petri dish, and specified concentrations of BER and AZM were added to the medium and covered in 15 mL melted agar, which was allowed to solidify. The Petri dish was dried at room temperature for 1 h. For swarming motility evaluation, a 5-*μ*L bacterial culture sample representing approximately10^8^ CFU/mL was inoculated on the center of the agar surface and cultured at 37°C for 48 h, then the diameters of the circular colonies were measured from the inoculation point. For twitching motility, a sterile toothpick was employed to inoculate one colony of bacterial culture deep into the agar of culture medium; subsequently, the motility at the agar-dish interface was measured.

### 2.9. Alginate Quantification

The specific methods used have been described [30]. Briefly, a single colony that had been cultured overnight was prepared in LB medium with no drug exposure and grown at 37°C for 24 h. The sample was pumped into a collection tube with a liquid container. The optical density (OD) of the sample at 600 nm (OD600) was measured by adding 1 mL of the solution to a disposable colorimetric plate and reading the OD, using a 722N spectrophotometer (Shanghai Precision Instrument Co., Ltd., Shanghai, China, 7058409084). Three replicate readings were obtained for each sample. The 50-*μ*L samples were added to an untreated 96-well microplate using a micropipette. Fifty microliters of carbonate-bicarbonate buffer at pH 9.6 was added to each well and the plate was incubated at 37°C for 2 h. Thereafter, PBS with 0.05% Tween 20 (PBS-T) (Shanghai Sangon Bioengineering Co., Ltd., Shanghai, China, G901DA0001) was used to wash the plate twice. A micropipette was used to add 200 *μ*L sealing buffer—10% skim milk in PBS-T—to each well, and the plate was incubated overnight at 4°C. Thereafter, PBS-T was used to rinse the plate twice. A micropipette was used to add 100 *μ*L diluted primary mouse anti-alginate monoclonal antibody to each well and the plate was incubated at 37°C for 1–2 h. Thereafter, the plate was washed thrice with PBS-T. A diluted secondary antibody (100 *μ*L) was added to each well and the plate incubated at 37°C for 1–2 h. The plate was again washed three times with PBS-T. A micropipette was then used to add 100 *μ*L tetramethylbenzidine (TMB)-ELISA solution from the TMB-ELISA kit (Shanghai Sangon Bioengineering Co., Ltd., Shanghai, China, G803DA0001) to the plate, before leaving it to incubate at 25°C in the dark, for 30 min. A total of 100 *μ*L termination solution was added with a micropipette and the Tecan enzyme-labelling instrument (Dana Tianjin Biological Technology Co., Ltd, Tianjin, China, 16039400) was used to read the OD value at 450 nm.

### 2.10. Quantitative Real-Time Polymerase Chain Reaction (qRT-PCR)

To verify the mRNA expression levels of alginate synthesis-related genes (*algG*, *algD*, and *algR*), Trizol reagent (Shanghai Sangon Bioengineering Co., Ltd., Shanghai, China, B511311-0100) was used to extract total RNA from PAO1 cultures that were either treated with BER and AZM or untreated. The concentration and purity of the extracted total RNA were determined using ultraviolet (UV) spectrometry at 260 and 280 nm. First-strand complementary DNA (cDNA) was synthesized from the extracted RNA by using a reverse transcription kit (Shanghai Sangon Bioengineering Co., Ltd., Shanghai, China, G709KA6194), per the manufacturer's instructions. The reverse-transcribed cDNA was stored at ˗20°C. RT-qPCR was performed using SYBR Green PCR Master Mix (Shanghai Sangon Bioengineering Co., Ltd., Shanghai, China, B639271-0005), with the primers listed in Table 1. Measurements were performed in triplicate. The housekeeping gene, *rpoB*, was used as an internal control, expression of the target gene was normalized, and the relative changes in gene expression were calculated. Expression levels of the alginate synthesis-related genes *algG*, *algD*, and *algR* were calculated using the threshold cycle (CT) method.

### 2.11. Statistical Analysis

All experiments were repeated at least three times to guarantee repeatability. Charts were constructed using GraphPad Prism software (https://www.graphpad.com/scientific-software/prism/; RRID: SCR_002798). Normality and variance homogeneity tests of each group of measurement data were conducted using SPSS Statistics for Windows, Version 20.0 software (IBM Corp., Armonk, NY, USA; RRID: SCR_019096). For data that showed normal distribution and uniform variance, Student's *t*-test was employed, whereas Welch's *t*-test was used for data with normal distribution and unequal variance. For abnormally distributed data, the Mann–Whitney *U* test was used.

## 3. Results

### 3.1. BER Enhances the Sensitivity of AZM Significantly *In Vitro*

The MIC of BER, AZM, and the combination of both against PAO1 was determined using the microdilution checkerboard method. As shown in Table 2, the MIC of the treatment agents against PAO1 was >1250.0 *μ*g/mL for BER, 256.0 *μ*g/mL for AZM, and 312.5–64.0 *μ*g/mL, in the case of the BER-AZM combination. The addition of BER significantly reduced the MIC of AZM, resulting in a synergistic effect. Therefore, the sub-MIC dose of the drug was used in subsequent experiments.

### 3.2. Time-Kill Assay

**Figure 1:**
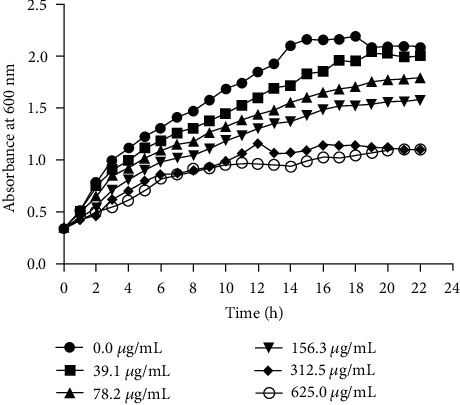
Time-kill curves of PAO1 cells post-treatments with different concentrations of BER.

As shown in Figure 1, the results showed that ≤78.0 *μ*g/mL BER did not affect the growth of PAO1, whereas 156.0 *μ*g/mL partially inhibited microbial growth. Moreover, at a concentration ≥312.5 *μ*g/mL, BER significantly decreased the growth rate and maximum growth level of PAO1, but could not completely kill the bacteria.

### 3.3. Effective Anti-Biofilm Activity of Berberine, Azithromycin, and the Combination of Both against *P. aeruginosa*

Considering the antibacterial efficacy of BER and AZM against *P. aeruginosa*, we investigated the potential anti-biofilm effect of BER, AZM, and the combination of both, against *P. aeruginosa* at subinhibitory concentrations. The results showed that subinhibitory concentrations of both BER and AZM indeed presented a certain measure of antibacterial membrane activity against PAO1. However, the combination of BER and AZM significantly reduced the formation of PAO1 biofilm (*P* < 0.01, vs. control group and BER, AZM alone treated group).

**Figure 2:**
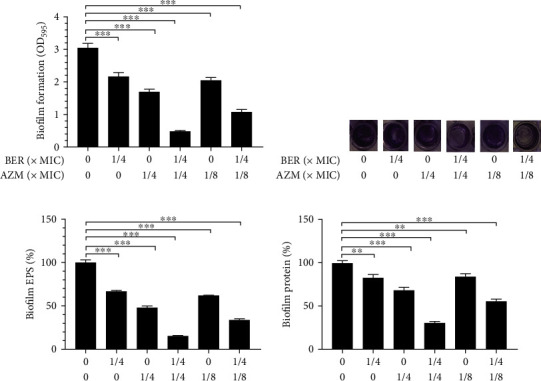
Effects of sub-MIC concentrations of BER and AZM on biofilm biomass, EPS, and total protein of PAO1. (a) The established biofilm formation (%) of PAO1 was measured by crystal violet staining at 595 nm post-treatments with different combinations of BER and AZM for 48 h at 37°C. (b) The visual image of biofilm colonized on microplates by crystal violet staining post-treatments with different combinations of BER and AZM for 48 h at 37°C. (c) Evaluations of biofilm EPS post-treatments with different combinations of BER and AZM for 48 h at 37°C. (d) Evaluations of biofilm protein post-treatments with different combinations of BER and AZM for 48 h at 37°C. Note: MIC: Minimum Inhibitory Concentration; BER: berberine; AZM: azithromycin; EPS: extracellular polysaccharide; ^∗^, *p* < 0.05; ^∗∗^, *p* < 0.01; ^∗∗∗^, *p* < 0.001; compared with the control group.

Due to the irreversible binding between biofilm microorganisms and the glass Petri dish surface, it is very difficult to extract all biofilm-related microorganisms from the glass surface. The traditional colony forming unit (CFU) method can only determine the number of microorganisms extracted from the biofilm and disregards unextracted microbial populations that remain in contact with the glass surface. Therefore, we adopted an indirect method for determining total biofilm protein levels, to illustrate the actual microbial abundance in the biofilm, as the number of extractable proteins is proportional to the number of attached microorganisms. In this study, we compared the EPS secretion and total protein levels between the drug-treated and untreated groups, as EPS and total protein are indicators of microbial biofilm formation. The results showed that BER and AZM in combination-treated bacteria secreted fewer EPS and total protein than the untreated bacteria, indicating that subinhibitory concentrations of the BER-AZM combination could effectively inhibit the development of *P. aeruginosa* biofilm. These results are shown in Figure 2.

### 3.4. BER and AZM in Combination Inhibit Elastase LasB and Pyocyanin Secretion by *P. aeruginosa*

**Figure 3:**
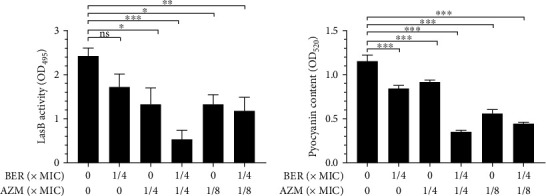
Effects of BER and AZM alone and in combination on the LasB and pyocyanin by PAO1. (a) Evaluations of LasB activity at OD495 post-treatments with different combinations of BER and AZM for 48 h at 37°C. (b) Evaluations of pyocyanin activity post-treatments with different combinations of BER and AZM for 48 h at 37°C. Note: MIC: Minimum Inhibitory Concentration; BER: berberine; AZM: azithromycin; ns: not statistically significant; ^∗^, *p* < 0.05; ^∗∗^, *p* < 0.01; ^∗∗∗^, *p* < 0.001; compared with the control group.

*P. aeruginosa* produces a variety of virulence factors, including elastase and pyocyanin. In this study, the effects of administering individual or combined subinhibitory concentrations of BER and AZM, against the secretion of elastase and pyocyanin by PAO1, were studied. As shown in Figure 3, subinhibitory concentrations of BER did not affect the production of Elastase LasB (*P* > 0.05, vs. control group), whereas AZM alone had some inhibitory effect (*P* < 0.05, vs. control group). However, the inhibitory effect of BER and AZM in combination was higher than that of any individual drug (*P* < 0.01, vs. control group). As for pyocyanin, both BER and AZM alone had significant inhibitory effects (*P* < 0.001, vs. control group). However, the combination of BER and AZM could inhibit the pyocyanin secretion more effectively than that of BER or AZM alone.

### 3.5. BER and AZM in Combination Inhibit the Swarming and Twitching Motility of PAO1

**Figure 4:**
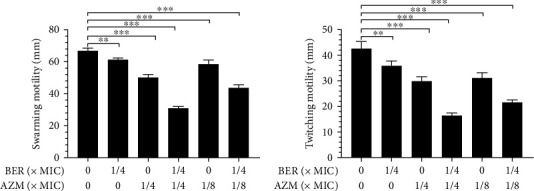
The effect of BER and AZM alone or in combination on swarming and twitching motilities of PAO1. (a) Evaluations of swarming motility post-treatments with different combinations of BER and AZM for 48 h at 37°C. (b) Evaluations of twitching motility post-treatments with different combinations of BER and AZM for 48 h at 37°C. Note: MIC: Minimum Inhibitory Concentration; BER: berberine; AZM: azithromycin; ns: not statistically significant; ^∗^, *p* < 0.05; ^∗∗^, *p* < 0.01; ^∗∗∗^, *p* < 0.001; compared with the control group.

The effects of BER and AZM, individually and in combination, on the swarming and twitching motility of *P. aeruginosa*, were determined after incubating PAO1 with the relevant drug concentrations, overnight. The results shown in Figure 4 reveal that subinhibitory concentrations of BER and AZM individually had zero and limited inhibitory effects on PAO1 swarming and twitching motility, respectively. However, the combination of the two drugs (BER-AZM: 312.5 *μ*g–64.0 *μ*g/mL) exerted a significant inhibitory effect on PAO1 swarming and twitching motility (*P* < 0.001, vs. control group).

### 3.6. BER and AZM in Combination Inhibit Alginate Secretion by PAO1 and Downregulate the Relative Expression of Alginate Formation-Related Genes of PAO1 Significantly

**Figure 5:**
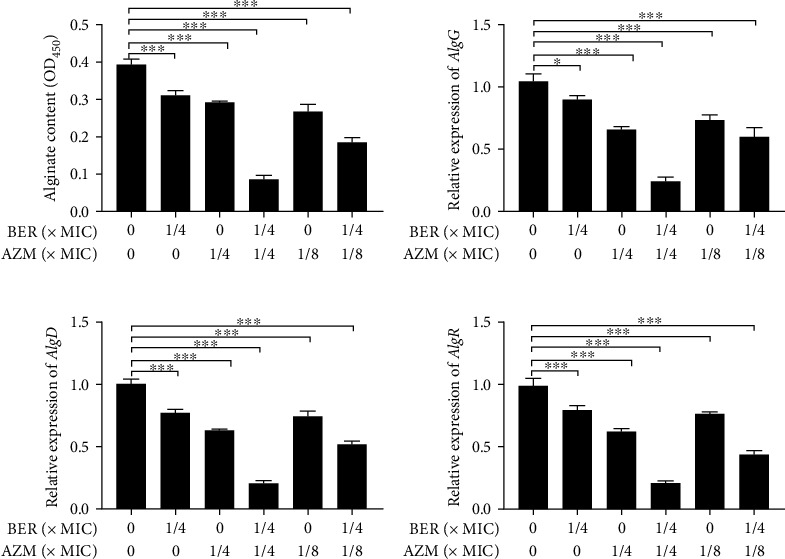
The inhibitory effect of BER and AZM alone and in combination on alginate and the expression levels of alginate-related regulatory genes of PAO1. (a) Analysis of alginate content at OD_450_. (b), (c), (d) Relative expressions of *AlgG*, *AlgD*, and *AlgR* by 2-*ΔΔ*Ct method, in which *ropB* was set as the reference control, post-treatments with the different combination at 37°C for 24 h. Note: MIC: Minimum Inhibitory Concentration; BER: berberine; AZM: azithromycin; ns: not statistically significant; ^∗^, *p* < 0.05; ^∗∗^, *p* < 0.01; ^∗∗∗^, *p* < 0.001; compared with the control group.

This evaluation was inspired by an experimental method related to the specific binding of alginate monoclonal antibodies to copper, as well as relative quantitative detection of alginate produced by PAO1. The quantitative results of alginate showed that the subinhibitory concentration of BER and AZM had a certain inhibitory effect on alginate production, which was significantly enhanced when the drugs were used in combination (*P* < 0.01, vs. control group), as shown in Figure 5.

The regulation, biosynthesis, and secretion of alginate are complex processes involving many adjacent structural genes, as well as regulatory factor-encoding genes such as *algR*, *algC*, *algB*, and *kinB* in *P. aeruginosa*. The biosynthesis and secretion of alginate EPS are initiated at the *algD* promoter, which encodes the first enzyme in the pathway leading to alginate biosynthesis, namely, GDP-mannose 6-dehydrogenase. In this study, the authors used qRT-PCR to detect the relative expression levels of *algR*, *algD*, and *algG* (an alginate C5-mannose isomerase in the alginate biosynthesis operon) in the PAO1. After 24 h of drug treatment, the relative expression levels of *algR*, *algD*, and *algG* of individually treated group (BER or AZM) all showed a certain extent of reduction (*P* < 0.001, vs. control group), except *AlgG* of 1/4 BER group (*P* < 0.05, vs. control group). When 1/4 MIC concentration of BER was added, the inhibitory effects of AZM upon alginate biosynthesis genes, including concentrations of 1/4 MIC and 1/8 MIC AZM, were significantly amplified. The results are shown in Figure 5.

## 4. Discussion

*P. aeruginosa* is a common opportunistic pathogen that accounts for chronic respiratory tract infection in patients with CF, and alginate is the main persistent factor following *P. aeruginosa* infection [31, 32]. The transformation of non-mucoid *P. aeruginosa* strains to alginate-overproducing mucoid strains in the early stages of infection in patients with CF is related to the decline in both lung function and survival rate [33]. At present, commonly used antibiotics in the clinic, such as cephalosporins, penicillins, carbapenems, and aminoglycosides, are frequently used in combination to treat *P. aeruginosa* infection. However, due to the increased incidence of multidrug-resistant *P. aeruginosa* strains in clinical settings, the anti-infection therapeutic effects of currently available antibiotics are quite limited [34]. Therefore, the organism has been classified as an ESKAPE group pathogen and listed by the World Health Organization as one against which new effective antimicrobial agents need to be developed urgently [35]. Among the resistance mechanisms of *P. aeruginosa*, antibiotic resistance caused by biofilms is predominant, and antimicrobial treatment without biofilm removal, which ultimately leads to repeated or persistent infections [36], is a presently serious cause of healthcare-associated concern [37].

Given the predominant role of biofilm in multidrug-resistant *P. aeruginosa*, the development of novel therapeutic strategies targeting biofilm is of the highest priority. Scientists have conducted many beneficial trials to evaluate the potential of artificial compounds [38], organic extracts [39], or modified compounds [40] from herbs, to combat such resistant strains. However, there is still a long journey to clinical application. BER is a quaternary ammonium alkaloid with a long history of medicinal application in Traditional Chinese Medicine, to treat intestinal infections such as gastroenteritis and bacterial dysentery [41]. Over the past several decades, various additional pharmacological effects of BER have been successively revealed by researchers. Studies using a *P. aeruginosa* pneumonia animal model have shown that BER plays an anti-inflammatory role by inhibiting the enrichment of leukocytes, reducing the inflammatory response induced by *P. aeruginosa*, and lowering the expression of pro-inflammatory factors [42]. In this study, the authors performed a series of *in vitro* experiments on the wild *P. aeruginosa* strain, PAO1. The results showed that BER could slightly inhibit the growth of PAO1 (MIC >1250.0 *μ*g/mL), whereas presented synergistic bacteriostatic activity when combined with AZM (Fractional Inhibitory Concentration Index =0.5). The results of the time-kill assay of BER against PAO1 also demonstrated that the sub-MIC levels (312.5 *μ*g/mL) of BER could dramatically inhibit the growth of this bacterial strain. Consequently, in the subsequent experiment, sub-MIC concentrations of BER were applied.

Biofilm facilitates the versatile lifestyle of *P. aeruginosa* and accounts for the pathogen's antibiotic resistance and bacterial immune evasion [43, 44]. Numerous studies have shown that bacterial cells encased by biofilm usually have a slower growth rate and increased secretion of various virulence factors, resulting in enhanced microbial pathogenicity [45, 46]. Therefore, it is imperative to adopt effective measures to prevent biofilm propagation and control the associated infection successfully. Previous studies reported the inhibitory effects of BER against biofilm and MexXY-OprM efflux pump of clinical strains of Pseudomonas aeruginosa to some extent [47–50]; however, PAO1 is allowedly considered a model microorganism for biofilm research and the results obtained from this strain are more persuasive. In this study, sub-MIC doses of the BER-AZM combination treatment considerably inhibited the biomass, EPS, and total protein of PAO1 biofilm. Moreover, the production of pyocyanin and LasB elastase, as well as bacterial motility, including swarming and twitching motility, were notably impeded by the combination of BER and AZM treatment. These data suggested that BER-AZM combination therapy could reduce the virulence and pathogenicity of *P. aeruginosa*, in vitro.

Alginate is the main component of secreted biofilm matrixes and the most striking feature of the mucoid *P. aeruginosa* [51, 52]. Herein, to evaluate the effects of the combination of BER-AZM upon alginate content in biofilm, an enzyme-linked immunosorbent assay (ELISA) method, using anti-alginate mouse monoclonal antibody, was employed to explore the effects of BER-AZM treatment against alginate production by PAO1. This method developed by the authors improved sensitivity and specificity compared with the traditional carbazole method [53]. Comparative analysis of alginate content produced by PAO1 treated with different combinations of BER-AZM indicated an optimal concentration (BER: 312.5 *μ*g/mL, AZM: 64.0 *μ*g/mL) that could notably down-regulated alginate biosynthesis. Simultaneously, relative expression of the alginate biosynthesis gene—*algD*—and corresponding regulatory genes, *algR* and *algG*, also showed a similar tendency. Nevertheless, whether the upstream pathway of alginate biosynthesis or the Quorum-sensing System was involved in the process requires intensive scientific investigation.

## 5. Conclusions

In summary, this study illustrated that the direct inhibitory activity of BER against PAO1 was limited. However, when combined with AZM, BER notably reduced the MIC of AZM and presented intensive synergistic antibacterial and -virulent activity. These findings provide firm proof for the immense potential of integration of Traditional Chinese Medicine and Western medicine in the therapy of infectious diseases caused by bacterial biofilm, despite intensive research being required in this respect.

## Figures and Tables

**Table 1:** Sequences of primers used for RT-qPCR.

Gene	Type	Primer sequence(5′-3′)	Tm (°C)	Length (bp)
*algG*	Forward Reverse	GCTGGTACGGCTTCTATTGCTACG ATCATCTTCTCCACGCCTAC	60.2 61.4	24 20
*algD*	Forward Reverse	AGAAGTCCGAACGCCACA TCCAGCTCGCGGTAGAT	56.8 56.0	18 17
*algR*	Forward Reverse	AGACCGGCTACGGCTACA GCGTCGTGCTTCTTCAGTT	58.8 55.9	18 19
*ropB*	Forward Reverse	AGGCCGTGAGCAGGGAT GGTGGTGCGACCGATGT	60.1 58.9	17 17

**Table 2:** Susceptibility of BER and AZM alone and in combination against planktonic PAO1.

Strain	MIC alone (*μ*g/mL)		MIC in combination (*μ*g/mL)		FICI (interpretation)
	BER	AZM	BER	AZM	
PAO1	>1250	256	312.5	64	0.5

Note: PA: *Pseudomonas aeruginosa*; MIC: Minimum Inhibitory Concentration; BER: berberine; AZM: azithromycin; FICI: Fractional Inhibitory Concentration Index.

## Data Availability

The authors confirm that the underlying data supporting the findings of this study are included within the article and its supplementary materials.

## Abbreviations

BER: Berberine
AZM: Azithromycin
CF: Cystic fibrosis
EPS: Extracellular polysaccharides
DNA: Deoxyribonucleic acid
ML: Macrolide
TCM: Traditional Chinese Medicine
LB: Luria-Bertani
ELISA: Enzyme-linked immunosorbent assay
CLSI: Clinical and Laboratory Standards Institute
PBS: Phosphate-buffered saline
ECR: Elastin-Congo red
CFU: Colony forming unit
MIC: Minimum inhibitory concentration
CT: Threshold cycle
FICI: Fractional inhibitory concentration index
qRT-PCR: Quantitative real-time polymerase chain reaction.

## Supplementary Materials

Supplementary Materials Scheme of the possible mechanisms of BER Combined with AZM against alginate production by PAO1.
