# Supplementary material for: In Vitro Synergistic Inhibitory Activity of Natural Alkaloid Berberine Combined with Azithromycin against Alginate Production by Pseudomonas aeruginosa PAO1
Source: Oxid Med Cell Longev. 2022 Sep 10;2022:3858500. doi: 10.1155/2022/3858500 (PMC9482538; doi:10.1155/2022/3858500)
Supplement: Supplementary Materials — Scheme of the possible mechanisms of BER Combined with AZM against alginate production by PAO1. [file 3858500.f1.docx]

**
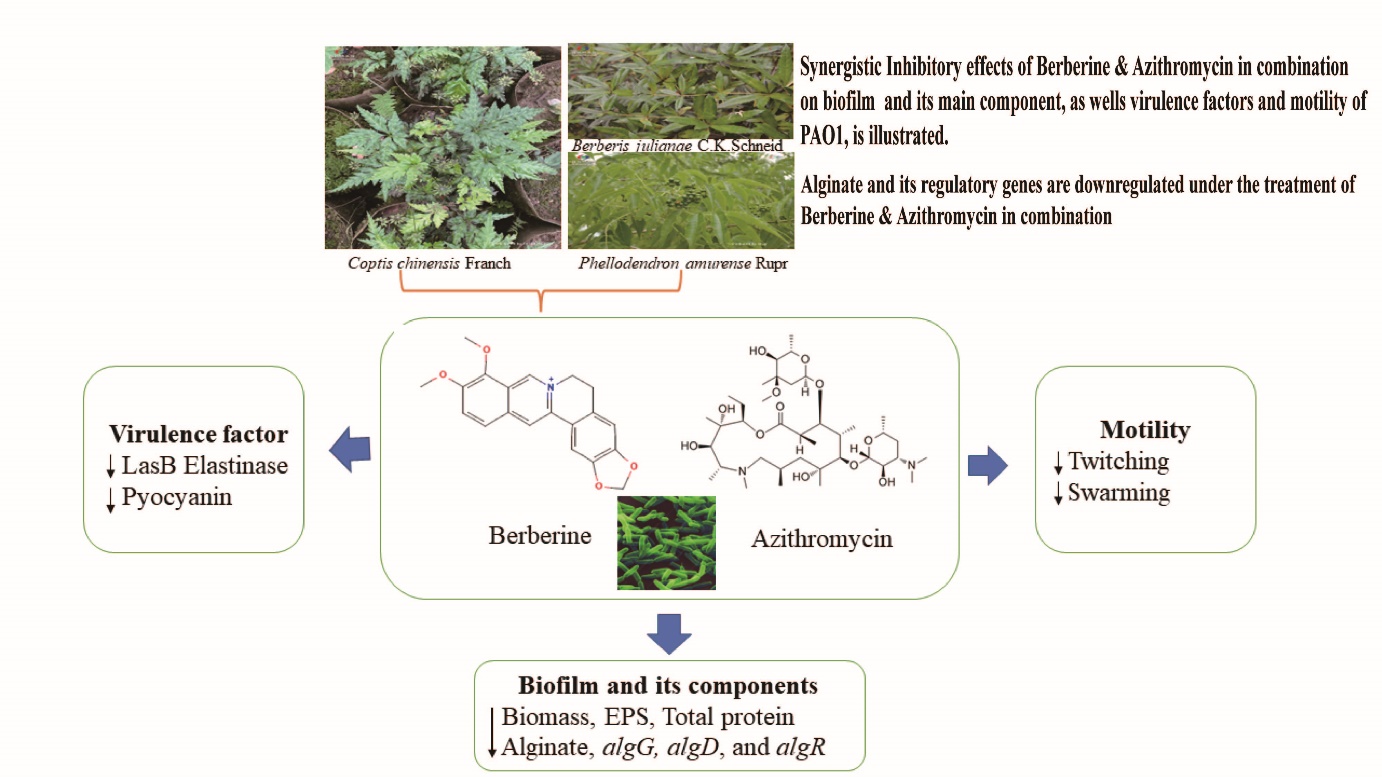
**

**SUPPLEMENTARY DESCRIPTION**

Scheme of the possible mechanisms of BER Combined with AZM against alginate production by PAO1
